# Species Composition and Ecological Aspects of Immature Mosquitoes (Diptera: Culicidae) in Phytotelmata in Cantareira State Park, São Paulo, Brazil

**DOI:** 10.3390/insects16040376

**Published:** 2025-04-02

**Authors:** Walter Ceretti-Junior, Antonio Ralph Medeiros-Sousa, Marcia Bicudo de Paula, Eduardo Evangelista, Karolina Morales Barrio-Nuevo, Ramon Wilk-da-Silva, Rafael Oliveira-Christe, Mauro Toledo Marrelli

**Affiliations:** 1 Department of Epidemiology, School of Public Health, University of São Paulo, São Paulo 01246-904, Brazil; cerettiw@usp.br (W.C.-J.); aralphms@yahoo.com.br (A.R.M.-S.); bicudo@usp.br (M.B.d.P.); entomol.edu@gmail.com (E.E.); karolinamorales182@gmail.com (K.M.B.-N.); ramonwilks@gmail.com (R.W.-d.-S.); 2 Health Surveillance Unit, São Paulo Municipal Secretariat, São Paulo 04262-000, Brazil

**Keywords:** phytotelmata, mosquitoes, biodiversity, mosquito-borne pathogens, Cantareira State Park

## Abstract

Phytotelmata are aquatic microenvironments formed by the accumulation of water and organic matter in cavities of plants. These microenvironments serve as breeding sites for various species of mosquitoes, including some of epidemiological importance. Our objective was to identify the mosquito fauna in these microenvironments and to analyze variations in mosquito fauna diversity between bromeliads, tree holes, and bamboo internodes in Cantareira State Park, São Paulo (CSP), Brazil, where there have been reports of yellow-fever epizootics in non-human primates and circulation of plasmodia. Collections were carried out monthly from February 2015 to April 2017. The bromeliads showed greater mosquito species richness and diversity than the tree holes and bamboo internodes, as well as a very different composition. Of the 11 genera collected and 49 taxa identified, *Culex* (*Carrolia*) *iridescens*, *Cx. ocellatus*, *Cx.* (*Microculex*) imitator, and *Anopheles* (*Kerteszia*) *cruzii* were the most abundant. The phytotelmata in the CSP showed significant differences in species richness, diversity, and composition and were found to support a diverse mosquito fauna to develop, including *An. cruzii* and the sylvatic yellow fever virus vector *Haemagogus leucocelaenus*. The finding of these epidemiologically important species highlights the key role played by phytotelma breeding sites as places of refuge and species maintenance for these vectors in green areas close to urban centers.

## Simple Summary

Several mosquito species, including some that spread disease-causing pathogens, use cavities, depressions, and other plant structures, where rainwater can accumulate, as breeding sites. This study compared the diversity of mosquito species found in bromeliads, tree holes, and bamboo internodes in Cantareira State Park, São Paulo, Brazil, an area with reported yellow fever outbreaks in monkeys and the presence of malaria-causing parasites. We collected immature mosquitoes over 27 months, identifying 49 species from 11 genera. The bromeliads had the highest number and variety of mosquito species among the phytotelmata studied. The results showed that these microenvironments can support a wide range of mosquito species, including *Anopheles cruzii*, an important malaria vector in the Brazilian Atlantic Forest, the sylvatic yellow fever virus carrier *Haemagogus leucocelaenus*, and *Aedes aegypti*, a vector of several arboviruses, including the dengue fever virus. These findings highlight the role of phytotelmata in maintaining mosquito populations, including disease-carrying species, in green areas near urban centers.

## 1. Introduction

Ecosystems have a complex plant physiognomy because of the biodiversity of species that comprise the flora and fauna in different biomes. Among the wide range of existing habitats and microhabitats, phytotelmata (from the Greek phyton = plant; telm = pond) are aquatic microenvironments formed by the retention of water in any part of the plant body, such as leaf axils, modified leaves, flowers, and holes in stems or fallen vegetative parts, including bracts, fruit peels, flowers, and inflorescences [1]. The water that accumulates in phytotelmata can originate from rain or metabolic processes, as observed in bamboo, resulting in a microenvironment with physical and chemical variations that can harbor different compositions of organisms [2,3]. These microenvironments are easy to handle, allowing studies and experiments to be carried out under natural conditions [4,5], and act as biotopes for various organisms, including terrestrial arthropods that have immature aquatic stages, such as mosquitoes (Diptera: Culicidae) [6,7]. By providing refuge for native and exotic populations of mosquitoes of epidemiological importance that act as vectors of pathogens, phytotelmata put the health of those handling them at risk [8,9].

Forming a taxonomic group of considerable biodiversity, with more than 3700 species described and validated to date, mosquitoes have adapted to the most diverse environments, from natural forests to highly urbanized areas [10]. Colonization in phytotelmata is selective and requires adaptations of the mosquito species that occupy them for oviposition and development of immature stages. Among the mosquito species found in these microenvironments, we highlight those of genera *Haemagogus*, *Trichoprosopon*, and *Sabethes* associated with tree holes and bamboo internodes [11]. In addition to occupying these two phytotelmata, some species of *Aedes* also appear to occupy bromeliads [12]. Mosquito species of subgenera *Kerteszia* of *Anopheles* and *Microculex* of *Culex*, as well as most species in the genus *Wyeomyia*, have greater affinity for bromeliads [13,14].

Most of the green areas in major cities have been converted to urban parks and environmental protected areas in order to preserve natural habitats and biodiversity and provide the population with leisure alternatives, space for physical activities, and the opportunity to have contact with nature. An example of these urban parks, Cantareira State Park (CSP), situated in the city of São Paulo, the most populous city in the southern hemisphere [15], provides important ecological services for greater São Paulo, such as the conservation of biodiversity and protection of aquatic environments by vegetation, as well as providing water for this region from springs in the park [16]. CSP also serves as a refuge for native and exotic fauna and flora, and there have been reports of enzootic cycles of vector-borne pathogens and epizootics of vector-borne diseases such as malaria and yellow fever (YF) involving mosquitoes and non-human primates in the park [17,18]. Both YF virus and the malaria-causing species in the genus Plasmodium that circulate in the Atlantic Forest are carried mainly by phytotelma mosquitoes of genera *Haemagogus* and *Anopheles*, respectively [19,20]. Recently, CSP was affected by yellow fever epizootics, which infected primarily the howler monkey population but also resulted in human cases [18,21].

Some studies have investigated the mosquito fauna of CSP [22,23,24,25,26,27]. A previous study conducted by our group aimed to provide an updated list of mosquito fauna in this park by employing various techniques to collect both adult and immature mosquitoes [25]. The results demonstrated that incorporating multiple mosquito capture methods led to an increase in the number of taxa collected, highlighting the importance of using diverse strategies to comprehensively sample the local mosquito fauna and explore a broader range of ecotopes [25]. We focused our analyses, taking into account the great importance of phytotelmata in the life cycle of mosquitoes. Therefore, the present work sought to identify the mosquito species found in phytotelmata in CSP and to analyze the variations in diversity of these insects according to the collection area and type of breeding site investigated.

## 2. Materials and Methods

### 2.1. Characterization of the Study Area: Cantareira State Park

**Figure 1:**
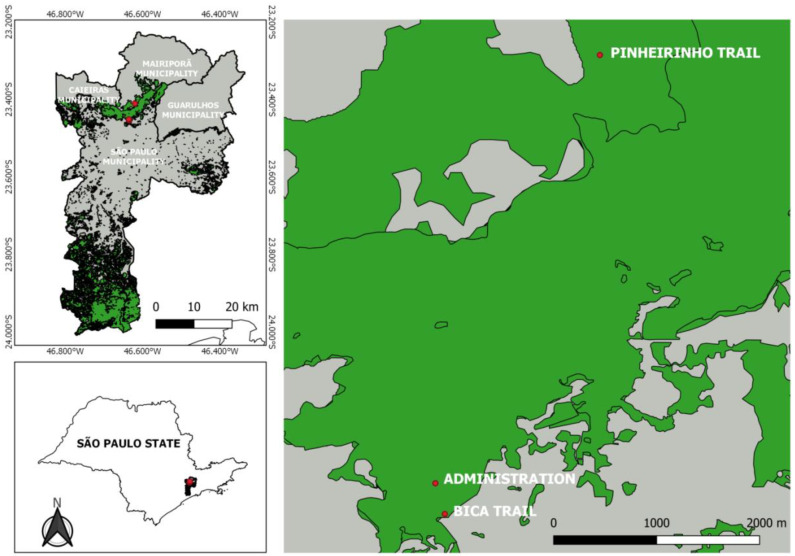
Locations of the sampled areas in Cantareira State Park; plant cover is highlighted in light green, while urban areas are highlighted in grey. The map was constructed using QGIS v3.4.12 (http://www.qgis.org). Source: Ceretti-Junior et al. [25].

With a total area of 7916.52 hectares, CSP is located in the northern region of the municipality of São Paulo between latitudes 23°35′/23°45′ S and 46°70′/46°48′ W, and extends into the municipalities of Mairiporã, Caieiras, and Guarulhos [15]. The climate in the region is classified as temperate and humid with wet summers and dry winters, with an average temperature of 22 °C or below in the warmest month and 14.3 °C in the coldest month. The rainy season is from October to March, when the average monthly rainfall is 186 mm, and the dry season is from April to September, when the average monthly rainfall is 51 mm. The average total annual rainfall is 1570 mm [15]. Considering the different combinations of major classes of landscape elements (urbanized, forest, transition area), three collection areas were selected to reflect the environmental gradient in the park (from urbanized to wild) (Figure 1).

Area 1 (Human-Impacted Environment)—Bica Trail (23°27.237′ S, 46°38.089′ W): This location was selected because of its proximity to dwellings close to a densely urbanized region with few green spaces. The trail is situated in an area featuring a uniformly structured, medium-height-to-tall forest canopy, which has experienced minimal man-made changes, despite being in a zone affected by human activities and invasion by domestic animals. The phytotelmata in this area consist of epiphytic bromeliads and some bamboo clumps.

Area 2 (Transition Area)—Administration Area (23°27.062′ S, 46°38.143′ W): Located deeper within the park, approximately 400 m from the interface between the forested area and the highly urbanized zone, the administration area has experienced significant changes. It features medium-height-to-tall trees with an uneven canopy structure, a grassy garden, two ponds, and some cultivated plants and trees with bromeliads, and is surrounded by native forest in the process of restoration.

Area 3 (Wild Environment)—Pinheirinho Trail (23°24.624′ S, 46°37.205′ W): This is a forested area with tall trees in an advanced stage of regeneration and with an uneven canopy structure. The area has not undergone many changes, and epiphytic bromeliads, tree holes, and bamboo internodes are still found in abundance. There is also a residential complex and country houses.

### 2.2. Mosquito Collection

Fieldwork was performed monthly from February 2015 to April 2017. All phytotelmata found within a radius of 200 m from a central point defined in each area were included in the sampling (soil bromeliads, epiphytic bromeliads, exposed bamboo internodes, bamboo internodes with lateral holes and tree holes). The phytotelmata were identified visually by the collectors, and those at heights of up to 18 m above the ground were explored. All identified phytotelmata that were accessible to the collectors and had water in their cavities were sampled and identified to be explored in subsequent field collections. Four bromeliads and eight bamboo internodes were identified and sampled in the Bica Trail. Seven bromeliads, two bamboo internodes, and one tree hole were sampled in the administration area. In the Pinheirinho Trail, eight bromeliads, four bamboo internodes, and three tree holes were sampled. To collect immature samples in the phytotelmata, the water was removed with manual suction pumps [26] and transferred to plastic containers, from which observed immature samples were collected with the aid of Pasteur-type plastic pipettes. The collected larvae and pupae were stored in 200 mL plastic bottles with part of the water from the breeding sites. The rest of the water (without immatures) was returned to the phytotelmata. The collected immature samples were transported to the Entomology Laboratory at the School of Public Health, University of São Paulo (LESP/FSP/USP), where they were kept until the emergence of the adults for morphological identification.

### 2.3. Identification and Cataloging

The species identification and cataloging was carried out at the LESP/FSP/USP. The species were identified morphologically with the taxonomic keys used by Lane [27], Galindo et al. [28], Corrêa and Ramalho [29], Arnell [30], Sirivanakarn [31], Consoli and Lourenço-de-Oliveira [32], and Forattini [33]. The abbreviations for genera and subgenera followed the standardization proposed by Reinert [34].

### 2.4. Statistical Analysis

The richness and diversity were compared between the study areas and between the different types of phytotelma breeding sites (bamboo, bromeliads, and tree holes) with the aid of rarefaction and extrapolation curves. To calculate the richness and diversity indices and construct the corresponding curves, the models proposed by Chao et al. [35] based on Hill’s numbers [36] were used. The diversity index used was Simpson’s reciprocal diversity index (1/D), and 95% confidence intervals for the estimates were calculated using the bootstrap method with 100 replicates. To reduce the effect of differences in sampling effort (the numbers of phytotelma breeding sites were different in the three areas), the rarefaction/extrapolation curves were based on the number of individuals rather than samples. To estimate and compare species richness between areas and between breeding site types, the curves were extrapolated up to the number of individuals observed in the area or breeding site type with the largest number of individuals, respectively. Similarly, to estimate Simpson’s reciprocal diversity index, the rarefaction curves were used to compare the different collection areas and breeding site types. In this case, the curves were estimated up to the number of individuals observed in the collection area or breeding site type with the lowest mosquito abundance.

To calculate the variation in species composition, two strategies were used. The first was to measure species dissimilarity between pairs of collection areas and breeding site types using Sorensen’s qualitative dissimilarity index. The second strategy was to measure the total dissimilarity in mosquito composition and partition this dissimilarity into nestedness (species loss or gain) and turnover (species replacement) [37]. Nestedness occurs when the species composition of an area is formed by a subset of the species composition of another area with greater richness, reflecting a non-random process of species loss or gain. Turnover occurs when species are replaced between environments, reflecting random processes or local characteristics that favor the colonization, permanence, or exclusion of certain species in each area. These two properties of beta diversity can be measured from the partitioning of Sorensen’s total dissimilarity index [38]. The analyses were performed in R 4.4.1 [38] with the iNEXT package [39].

## 3. Results

### 3.1. General Results

A total of 3124 immature Culicidae specimens belonging to 49 taxa distributed in 11 genera (*Aedes*, *Anopheles*, *Culex*, *Haemagogus*, *Lutzia*, *Sabethes*, *Toxorhynchites*, *Trichoprosopon*, *Shannoniana*, *Runchomyia*, and *Wyeomyia*) (Table S1) were collected. Of these, the genus *Culex* (2048 specimens, 65.56% of the specimens collected) was the most representative, comprising 22 taxa (corresponding to 44.90% of the taxa sampled) in 4 subgenera, of which the subgenus *Microculex* was the richest (14 taxa), followed by the genera *Wyeomyia* (11 taxa) and *Toxorhynchites* (7 taxa).

Among the most abundant species collected, we can highlight *Cx.* (*Car*.) *iridescens* (*n* = 547), *Cx. ocellatus* (*n* = 308), *Cx*. (*Mcx.*) *imitator* (*n* = 301), *An.* (*Ker.*) *cruzii* (*n* = 257), *Cx.* (*Mcx.*) *pleuristriatus* (*n* = 191), *Wy.* (*Pho.*) *theobaldi* (*n* = 204), *Cx.* (*Mcx.*) *worontzowi* (*n* = 155), *Sh. fluviatile* (*n* = 145), *Cx.* (*Mcx.*) *albipes* (*n* = 124), *Wy.* (*Pho.*) *davisi* (*n* = 123), and *Cx.* (*Mcx.*) sp. (*n* = 116), which together accounted for about 79.10% of the total number of specimens collected (Table S1).

The following species occupied all three sampling sites we explored: *An.* (*Ker*.) *cruzii*, *Cx.* (*Mcx*.) *albipes*, *Cx.* (*Mcx*.) *imitator*, *Cx.* (*Mcx*.) *pleuristriatus*, *Cx.* (*Mcx*.) *worontzowi*, *Cx. ocellatus*, *Wy.* (*Pho.*) *davisi*, and *Wy.* (*Pho.*) *theobaldi*. Only *Cx. ocellatus* was found in all three types of phytotelma we explored.

In terms of epidemiologically important species, *An. cruzii* was the most abundant, with 257 individuals collected (8.23% of the total), all of which were collected in bamboo plants, with 82% of the individuals collected on the Pinheirinho Trail. *Hg. leucocelaenus* had 71 individuals (2.27%) collected in similar proportions between tree holes (52%) and bamboo (48%), with 87% of the individuals collected on the Pinheirinho Trail. *Ae. aegypti* had 29 individuals collected (0.93%) and was found in all three types of phytotelmata but mainly in bamboo (75%); the species was present on the Bica Trail and the Pinheirinho Trail. In turn, only 14 individuals of *Ae. albopictus* were collected (0.45%) in both bamboo plants (71%) and bromeliads, and this species was observed in all three study areas (Table S1). All four species were collected in greater numbers during the warm and rainy periods (from November to April) (Table S2).

When the temporal distribution of these specimens by collection date was analyzed (Table S2), it was observed that some species, such as *An. cruzii*, *Cx. iridescens*, *Cx. imitator*, *Cx. worontzowi*, *Cx. ocellatus*, *Wy. davisi*, and *Wy. theobaldi*, were quite frequent in terms of the number of times they were collected during the study and were among the most abundant species, while others were infrequent or extremely infrequent. December 2015 and January and February of 2016 were the months with the greatest abundance and richness of mosquitoes, coinciding with the rainy summer months.

### 3.2. Richeness and Diversity by Area

**Figure 2:**
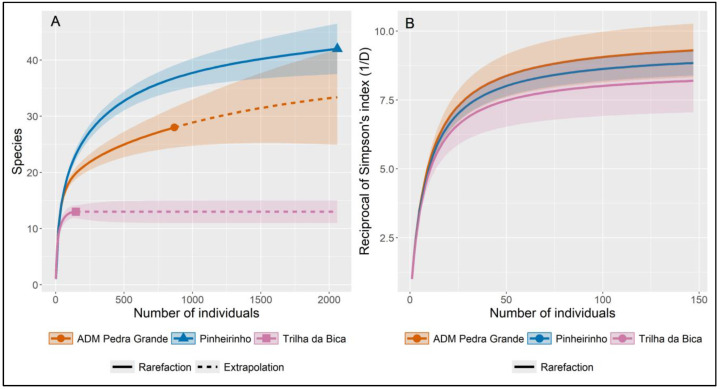
Rarefaction and extrapolation curves (with 95% CI) of (**A**) species richness and (**B**) Simpson’s reciprocal diversity index (1/D) for mosquitoes collected in phytotelmata at three Cantareira State Park collection areas. The shaded areas represent the 95% confidence interval for the mean estimate.

Of the areas investigated, the Pinheirinho Trail yielded the highest number of specimens collected (65.6% of the total) and the highest number of taxa (42), followed by the administration area (27% of the collected specimens and 28 taxa). Simpson’s reciprocal diversity index varied little between the environments and was higher in the administration area and lower in the Bica Trail, with overlapping confidence intervals (Table 1 and Figure 2).

### 3.3. Richeness and Diversity by Phytotelmata

**Figure 3:**
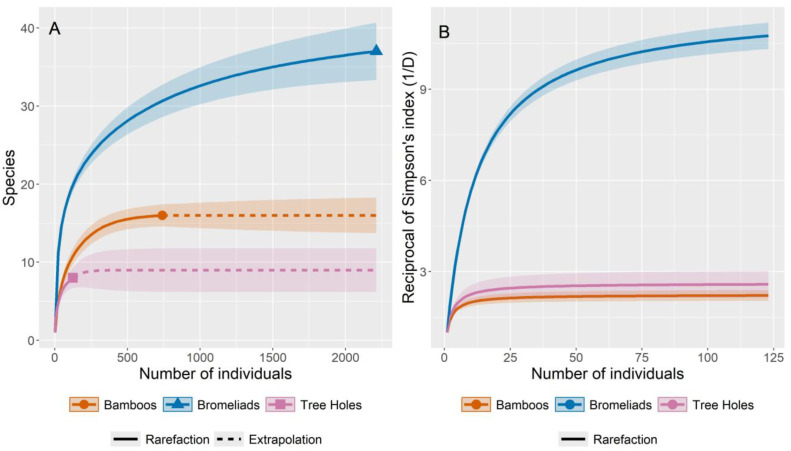
Rarefaction and extrapolation curves (with 95% CI) of (**A**) species richness and (**B**) Simpson’s reciprocal diversity index (1/D) for Culicidae collected in three types of breeding sites (phytotelmata) in Cantareira State Park (CSP). The shaded areas represent the 95% confidence interval for the estimated mean.

Our results show that bromeliads had the highest numbers of both specimens and species, followed by bamboo internodes. The richness estimates suggest that with an increase in sampling effort, more species could have been collected in the bromeliads and tree holes. The Simpson’s reciprocal diversity index values varied greatly between the different types of breeding sites and was high for bromeliads and relatively low for bamboo and tree holes (Table 2 and Figure 3).

### 3.4. Dissimilarity Between Areas and Phytotelmata

The dissimilarity in species composition between the collection areas had an intermediate value (0.53), while the corresponding figure between the breeding site types was relatively high (0.79) (Table S3). The greatest dissimilarity between locations was between the Bica Trail and the other areas (>0.5) (Table S4), while the dissimilarity between breeding site types was quite high between bromeliads and tree holes (0.82) and bromeliads and bamboo (0.77) (Table S5). By partitioning the dissimilarity between nestedness and turnover, it was observed that whereas the variation between study areas had a similar contribution from each of these processes, the variation between breeding site types was mainly due to species turnover (Table S3).

## 4. Discussion

Phytotelmata are excellent breeding sites for mosquitoes, which exhibit notorious plasticity in their colonizing habits and coexistence with other animal groups in these microenvironments [6,7,40]. This is in agreement with our results, which reveal a high diversity of Culicidae breeding in these natural ‘containers’. Among the mosquito species found in the phytotelmata in CSP were some of major epidemiological importance, such as *Hg. leucocelaenus*, *An. Cruzii*, and *Ae. aegypti*. Our analyses show that the composition of the species in these microhabitats varies more between the different types of breeding sites than between the three areas studied. The richness estimate suggests that with an increased sampling effort, more species could have been collected in the administration area and Pinheirinho Trail. The bromeliad breeding sites contributed the most to species richness and exhibited significantly higher diversity indices than the bamboo and tree holes. Additionally, the species composition in the bromeliads differed markedly from the tree holes and bamboo internodes. Many factors may have contributed to this result, such as the sunlight exposure, water volume, and organic materials accumulated in leaf axils, which form a unique microcosm for several insect species [8]. Although the Simpson’s reciprocal diversity index did not vary significantly between the study areas, the collection area with the highest species richness was the most preserved of the three, and the area with the lowest number of species was the most urbanized, corroborating the results of previous studies [25,41,42].

The largest numbers of species found were in the genera *Culex* (mainly in the subgenus *Microculex*) and *Wyeomyia*, which are commonly recorded in surveys of mosquito fauna in bromeliads, tree holes, and bamboo internodes, supporting the findings of previously published studies [40,43,44,45]. *Culex* is the genus with the largest number of species described to date; 819 species distributed in 27 subgenera have been identified [10], partly explaining the species richness for this genus observed in the present study. The genus is cosmopolitan and generally colonizes permanent underground water bodies. However, many *Culex* species colonize holes in rocks, holes made by crabs, phytotelmata, or even artificial containers [10,46].

The presence of *Cx.* (*Car.*) *iridescens*, *Cx. ocellatus*, *Cx.* (*Mcx.*) *imitator*, *Cx.* (*Mcx.*) *pleuristriatus*, and *Cx.* (*Mcx.*) *albipes* among the species collected in this work, which was conducted in an area recognized as one of the largest urban forests in the world, can be attributed to the fact that these mosquito species are highly adapted to wild environments [47,48], although certain species in the *Microculex* subgenus are also found in environments where vegetation loss is significant [48].

The finding of species of the subgenus *Carrollia* is not surprising because this subgenus includes 18 wild species found in neotropical forests in southeastern Mexico, Brazil, and northeastern Argentina, which are considered to have high ecological plasticity and to be associated with several breeding sites, including broken and cut bamboo [49,50,51].

The *Culex ocellatus* and *Culex* (*Microculex*) species recorded here are highly dependent on bromeliads for their larval habitat [9,52,53]. The former is abundant in bromeliads in both floodplain areas and mountains in the Atlantic Forest in less sunny areas [9,12,44]. In the present study, *Cx. ocellatus* was observed in all three environments and in all phytotelmata explored. Although species of the subgenus *Microculex* are usually found in preserved environments [43], some species of this subgenus collected in this study, such as *Cx.* (*Mcx.*) *pleuristriatus* and *Cx.* (*Mcx.*) *albipes*, exhibit ecological plasticity and can use artificial breeding sites in urban and periurban areas [40,48].

The high number of specimens of *An.* (*Ker.*) *cruzii* collected in the present study can be attributed to the fact that epiphytic bromeliads were the most abundant phytotelma in the areas surveyed. These plants serve as natural breeding sites for this species of mosquito, which is involved in the transmission of the etiological agents of human and simian malaria in the southern and southeastern regions of Brazil, where bromeliaceae are abundant, especially in primary forests [54,55,56]. In contrast, *An.* (*Ker.*) *bellator* was rare, and only one specimen was found during the entire collection period. The finding of *An. cruzii* in the three areas explored can be explained by the fact that the subgenus *Kerteszia* is considered a bioindicator of preserved environments [57]. However, members of this subgenus have highly anthropophilic behavior, and the finding of these mosquitoes in human households has long been reported [58], which explains their occurrence in the Bica Trail, an environment in close proximity to human dwellings.

Immature specimens of *Wy.* (*Pho.*) *davisi* and *Wy.* (*Pho.*) *theobaldi* are commonly found in bromeliads, and adults are usually found in humid forests close to larval breeding sites. Adult females have been observed feeding on humans and other animals, and were also collected in traps using birds as bait [59,60]. Although they bite humans, there are no reports of these species participating in viral transmission cycles. With the exception of *Wy.* (*Pho.*) *trinidadensis*, all other species of the subgenus *Phoniomyia* occur in Brazil, and only six species of this group have been observed outside Brazil [10].

Our finding of species of *Cx.* (*Car*.) and *Cx*. (*Mcx*.), *Wyeomyia*, *Shannoniana*, and *Toxorhynchites* attests to the good state of preservation of the environments explored in CSP. These Culicidae are considered to belong to the natural mosquito fauna in the state of São Paulo and are better adapted to wild environments [40,61,62].

Immature specimens of *Ae. aegypti* were collected in the three types of phytotelmata, while specimens of *Ae. albopictus* were collected only in bromeliads and bamboo and in smaller numbers. *Aedes aegypti* is considered highly anthropophilic and synanthropic, and is very dependent on artificial containers for breeding [63]. *Aedes albopictus*, in turn, is more associated with wild environments and occupies different forest habitats but also rural and periurban human-impacted environments, where it has a tendency to explore peridomestic environments during the day [64]. While immature forms of *Ae. aegypti* and *Ae. albopictus* have long been recorded in bromeliads, in particular in environments changed as a result of human activities [12,65], they also occur in preserved environments but always in low densities in relation to other Culicidae species, such as *Culex* belonging to the *Microculex* and *Ocellatus* group and species of *Phoniomyia* of *Wyeomyia* that appear to be closely associated with bromeliads in the neotropics [12]. Thus, it seems that the interspecific competition with mosquitoes specialized in bromeliads makes these habitats less favorable to generalist species that use artificial breeding sites such as *Ae. albopictus* and *Ae. aegypti*, corroborating the results of previous studies [12,66,67].

The finding of *Hg. leucocealeanus* in CSP corroborates the results reported by Mucci et al. [24] and Wilk-da-Silva et al. [68,69]. This species typically colonizes tree holes, although it is also found in artificial breeding sites [70,71,72].

Our results show fewer taxa in tree holes and bamboo than in bromeliads. Tree holes and bamboo are among the oldest and most specialized breeding sites for mosquito larvae [14]. They are also the primary breeding sites for *Aedes* mosquito species such as *Ae. aegypti* in Africa, *Ae. albopictus* in Asia, and *Ae. serratus*, all of which are implicated in the transmission of the YF virus [73,74,75,76], as well as other important arboviruses such as dengue virus (DENV), chikungunya virus (CHIKV), and Zika virus (ZIKV) [77].

A study conducted by Multini et al. [78] in CSP evaluating the physicochemical parameters of mosquito breeding sites showed that bromeliads had a greater variation in pH than bamboo and tree hollows, while the latter showed a greater variation in salinity compared to the bromeliads. These variations in water properties may explain the high turnover and variation in species richness between the types of phytotelmata investigated, as the capacity for ionic and osmotic regulation varies between different mosquito species.

Bamboo is used for landscaping purposes in parks but also occurs naturally, and one of the largest native bamboo forests on the planet is in the southern Amazon [79]. Their internodes can be used as breeding sites when the stem breaks transversely and the internode is exposed to rainwater, making it a suitable habitat for oviposition and the development of immature mosquitoes [14]. In addition, wild insects such as some coleoptera can drill laterally into the stems of these plants, allowing rainwater and physiological water to accumulate in the internodes, which become potential habitats for mosquito larvae [3,14,80]. The mosquito species found in bamboo culms are considered wild and develop their own strategies to exploit these specialized environments [14,80].

Our results show that bromeliads were the most important phytotelmata in the study, with a greater richness of species collected compared with the bamboo and tree holes, as they constitute a permanent source of water and micronutrients and are, therefore, productive breeding grounds for mosquitoes [9]. These results disagree with those reported by Muller et al. [81], who found that bamboo plants were richer in immature forms than other phytotelmata. In previous studies by our group carried out in fragments of the Atlantic Forest in urban parks in São Paulo, we observed lower mosquito richness levels in naturally perforated bamboo than in other natural breeding sites such as bromeliads and lakes [2]. Our results also corroborate the results of Bastos et al. [82], who observed greater species richness in bromeliads than in bamboo and lakes.

Of all the Bromeliacea species, 40% are found in Brazil, mostly in the Atlantic Forest, where they are considered one of the main breeding sites for wild species of Culicidae. More than 30 species of this family have been observed in water accumulated in the leaf axils of these plants [44,81,83], and in the present study we found 37 species of Bromeliacea in CSP alone. Bromeliads are also widely used for ornamentation in public and private gardens and can be observed in the natural landscapes of ecologically preserved areas, as well as in residential gardens and urban parks in the municipalities within the Atlantic Forest biome [9,61].

Phytotelmata are important natural breeding sites capable of retaining water and organic matter and maintaining ecological relationships between organisms that inhabit these microenvironments. Therefore, they can serve as shelters and refuges for some autochthonous and exotic species of mosquito fauna, which have been shown to transmit etiological agents of disease to humans and other animals. The presence of *Ae. aegypti* and *Hg. leucocelaenus* in CSP points to a permanent scenario involving a risk of spillover of the YF virus from the wild to the urban environment, whereas the occurrence of *An. cruzii* is closely associated with the maintenance of simian malaria transmission within the park.

## Figures and Tables

**Table 1:** Observed and estimated values of species richness and Simpson’s reciprocal diversity index (1/D) for phytotelma Culicidae collected in the three study areas in Cantareira State Park (CSP), including upper and lower estimates for 95% CI.

Diversity Index	CSP Collection Area		
	Administration Area	Pinheirinho Trail	Bica Trail
Individuals	868	2059	148
Observed richness	28	42	13
Estimated richness	37	60	13
Upper Est_richness	58	82	15
Lower Est_richness	28	42	13
Observed Simpson	9.76	9.31	8.20
Estimated Simpson	9.86	9.34	8.62
Upper Est_Simpson	10.81	9.92	9.83
Lower Est_Simpson	8.91	8.77	7.41

**Table 2:** Observed and estimated values for species richness and Simpson’s reciprocal diversity index (1/D) for Culicidae collected in three types of breeding sites (phytotelmata) in Cantareira State Park (CSP), including upper and lower estimates for the 95% CI.

Diversity Index	Breeding-Site Type		
	Bamboo	Bromeliads	Tree Holes
Individuals	739	2212	124
Observed richness	16	37	8
Estimated richness	16	43	9
Upper Est_richness	20	57	12
Lower Est_richness	16	37	8
Observed Simpson	2.24	11.63	2.59
Estimated Simpson	2.24	11.69	2.62
Upper Est_Simpson	2.43	12.18	3.07
Lower Est-Simpson	2.06	11.19	2.17

## Data Availability

The original contributions presented in this study are included in the article/Supplementary Materials. Further inquiries can be directed to the corresponding authors.

## Supplementary Materials

The following supporting information can be downloaded at https://www.mdpi.com/article/10.3390/insects16040376/s1, Table S1. Culicidae immature samples collected in phytotelmata in Cantareira State Park, São Paulo, Brazil from February 2015 to April 2017. Table S2. Temporal distribution of Culicidae immature forms collected in phytotelmata in the Cantareira State Park, São Paulo, Brazil, according to the month of collection, from February 2015 to April 2017. Table S3. Total and partitioned dissimilarity index (turnover and nestedness) values for phytotelma mosquito composition for the different study areas (administration area, Pinheirinho Trail, and Bica Trail) and different types of breeding sites (bromeliads, bamboo, and tree holes). Table S4. Dissimilarity of phytotelma Culicidae species compositions in the three CSP collection areas measured with the Sorensen index. Table S5. Dissimilarity of phytotelma Culicidae species compositions for the three breeding site types measured with the Sorensen index.
