# Supplementary material for: Species Composition and Ecological Aspects of Immature Mosquitoes (Diptera: Culicidae) in Phytotelmata in Cantareira State Park, São Paulo, Brazil
Source: Insects. 2025 Apr 2;16(4):376. doi: 10.3390/insects16040376 (PMC12027585; doi:10.3390/insects16040376)
Supplement: Supplementary file 1 [file insects-16-00376-s001.zip › SupplementarMaterial-2.pdf]

Table S3. Total and partitioned dissimilarity index (turnover and nestedness) for phytotelma mosquito composition for the different study areas (Administration Area, Pinheirinho Trail and Bica Trail) and different types of breeding sites (bromeliads, bamboo and tree holes).

**Index**	**CSP collection area**	**Breeding-site type**
Total dissimilarity	0.53	0.79
Turnover	0.23	0.58
Nestedness	0.3	0.21

Table S4. Dissimilarity of phytotelma Culicidae species composition in the three CSP collection areas measured with the Sorensen index.

	**Sorensen dissimilarity index**	
	Administration Area	Pinheirinho Trail
Pinheirinho Trail	0.4	
Bica Trail	0.51	0.53

Table S5. Dissimilarity of phytotelma Culicidae species composition for the three breeding-site types measured with the Sorensen index.

	**Sorensen dissimilarity index**	
	Bromeliads	Bamboo
Bamboo	0.77	
Tree Holes	0.82	0.58
