# Supplementary material for: Species Composition and Ecological Aspects of Immature Mosquitoes (Diptera: Culicidae) in Phytotelmata in Cantareira State Park, São Paulo, Brazil
Source: Insects. 2025 Apr 2;16(4):376. doi: 10.3390/insects16040376 (PMC12027585; doi:10.3390/insects16040376)
Supplement: Supplementary file 1 [file insects-16-00376-s001.zip › Table S1.pdf]

Table S1. Culicidae immatures collected in phytotelmata in Cantareira State Park, São Paulo, Brazil from February 2015 to April 2017.

Taxon	Administration			Pinheirinho Trail			Bica Trail				
	1	2	3	1	2	3	1	2	3	Total	%
Aedes (*Stegomyia*) *aegypti aegypti* (Linnaeus, 1762)				3		4		22		29	0.93
Aedes (*Stegomyia*) *albopictus* (Skuse, 1895)	4	4			3			3		14	0.45
Anopheles (*Kerteszia*) *bellator* Dyar & Knab 1906	1									1	0.03
Anopheles (*Kerteszia*) *cruzii* Dyar & Knab, 1908	39			210			8			257	8.23
Culex (*Carrollia*) *iridescens* (Lutz, 1905)				18	451	5		73		547	17.51
Culex (*Carrollia*) sp.					19					19	0.61
Culex (*Culex*) *dolosus* (Lynch Arribálzaga, 1891)	7		36			31				74	2.37
Culex (*Culex*) *eduardoi* Casal & García, 1968					1	6				7	0.22
Culex (*Microculex*) *albipes* Lutz, 1904	35			77			12			124	3.97
Culex (Microculex) dubitans Lane & Whitman, 1951				16						17	0.54
Culex (*Microculex*) *imitator* Theobald, 1903	80			189	5		27			301	9.64
Culex (*Microculex*) *imitator daumasturus* Dyar & Knab 1906				1						1	0.03
Culex (*Microculex*) Imitator group	46			33						79	2.53
Culex (*Microculex*) inimitabilis *fuscatus* Lane & Whitman, 1951				5						5	0.16
Culex (*Microculex*) *inimitabilis inimitabilis* Dyar & Knab, 1906				6						6	0.19
Culex (*Microculex*) *lanei* Oliveira Coutinho & Forattini, 1962	32									32	1.02
Culex (*Microculex*) *neglectus* Lutz, 1904	1			13						14	0.45
Culex (*Microculex*) *pleuristriatus* Theobald, 1903	157			9			25			191	6.11
Culex (*Microculex*) *pleuristriatus/albipes*				7						7	0.22
Culex (*Microculex*) Pleuristriatus group	30			7						37	1.18
Culex (Microculex) *reducens* Lane & Whitman, 1951	5									5	0.16
Culex (*Microculex*) sp.	90			26						116	3.71
Culex (*Microculex*) *worontzowi* Pessoa & Galvão, 1936	19			128			8			155	4.96
Culex (*Melanoconion*) *intrincatus* Brèthes, 1916				3						3	0.10
Culex ocellatus Theobald, 1903	20			267	13	1	7			308	9.86
Haemagogus (*Conopostegus*) *leucocelaenus* Dyar & Shannon, 1924		1			25	37		8		71	2.27
Lutzia (*Lutzia*) *bigoti* (Bellardi, 1862)						1				1	0.03
Runchomyia (*Runchomyia*) *cerqueirai* Stone 1944					7					7	0.22
Sabethes (*Sabethes*) *purpureus* (Theobald, 1907)						3				3	0.10
Shannoniana fluviatile (Theobald, 1903)					145					145	4.64
Trichoprosopon pallidiventer (Lutz, 1905)					27					27	0.86
Toxorhynchites (*Ankylorhynchus*) *trichopygus* (Wiedemann) 1928	3									3	0.10
Toxorhynchites (*Lunchella*) *theobaldi/moengoensis*				2						2	0.06
Toxorhynchites bambusicola aff.				1						1	0.03
Toxorhynchites guadeloupensis aff..	2									2	0.06
Toxorhynchites trichopygus aff.	2									1	0.03
Toxorhynchites purpureus/catharinensis	2			1						3	0.10
Toxorhynchites sp.	9			11			2	1		23	0.74
Wyeomyia personata					4					4	0.13
Wyeomyia (*Phoniomyia*) *davisi* (Lane & Cerqueira, 1942)	32			82			9			123	3.94
Wyeomyia (*Phoniomyia*) *edwardsi* (Lane & Cerqueira, 1942)	23			1						24	0.77
Wyeomyia (*Phoniomyia*) pallidoventer Theobald 1907	19			42						61	1.95
Wyeomyia (*Phoniomyia*) *palmata* (Lane & Cerqueira, 1942)				3						3	0.10
Wyeomyia (*Phoniomyia*) sp.	15			33						48	1.54
Wyeomyia (Phoniomyia) theobaldi (Lane & Cerqueira, 1942)	129			49	3		23			204	6.53
Wyeomyia. sp.					4					4	0.13
Wyeomyia (Spilonympha) airosai Lane & Cerqueira, 1942										1	0.03
Wyeomyia (*Wyeomyia*) *oblita* Lutz 1904					3			2		5	0.16
Wyeomyia (*Wyeomyia*) *lutzi* (Costa Lima) 1930					9					9	0.29
Total	**803**	**5**	**36**	**1,243**	**719**	**88**	**121**	**104**	**0**	**3,124**	100.0

1 - Bromeliads; 2 – Bamboo; 3 – Tree Holes.
