# Supplementary material for: Species Composition and Ecological Aspects of Immature Mosquitoes (Diptera: Culicidae) in Phytotelmata in Cantareira State Park, São Paulo, Brazil
Source: Insects. 2025 Apr 2;16(4):376. doi: 10.3390/insects16040376 (PMC12027585; doi:10.3390/insects16040376)
Supplement: Supplementary file 1 [file insects-16-00376-s001.zip › Table S2.pdf]

Table S2. Temporal distribution of Culicidae immature forms collected in phytotelmata in the Cantareira State Park, São Paulo, Brazil, according to the month of collection, from February 2015 to April 2017.

Taxa / Month-Year	2015											2016												2017				
	Feb	Mar	Apr	May	Jun	Jul	Aug	Sep	Oct	Nov	Dec	Jan	Feb	Mar	Apr	May	Jun	Jul	Aug	Sep	Oct	Nov	Dec	Jan	Feb	Mar	Apr	
Ae. (Stg.) aegypti														19		5							1			3	1	**29**
Ae. (Stg.) albopictus												4	4		3												3	**14**
An. (Ker.) bellator			1																									**1**
An. (Ker.) cruzii	4	15	4	24	26	7	2	18		3	96	7	16	4		10	6	3				3	2	5		1	1	**257**
Cx. (Car.) iridescens								22	12	40	39	101	92	68	36	2	18	18		8	26		12	9	9	18	16	**547**
Cx. (Car.) sp.			6	13																								**19**
Cx. (Cux.) dolosus								7		1	30	36																**74**
Cx. (Cux.) eduardoi																										3	4	**7**
Cx. (Mcx.) albipes		4	7		21	11		28			34	11	8															**124**
Cx. (Mcx.) daumasturus													1															**1**
Cx. (Mcx.) dubitans										5		6	5	1														**17**
Cx. (Mcx.) fuscatus													4							1								**5**
Cx. (Mcx.) Imitator group		57	17	5																								**79**
Cx. (Mcx.) Pleuristriatus group	7	20		10																								**37**
Cx. (Mcx.) imitabilis					6																							**6**
Cx. (Mcx.) imitator	19		4	1	7	11	3	70		21	24	39	71	12	1		1	9		1		1	1	5				**301**
Cx. (Mcx.) lanei		31	1																									**32**
Cx. (Mcx.) neglectus				8				3					1			1		1										**14**
Cx. (Mcx.) pleuristriatus	29				4						14	31	16	9			6	15						54		4	9	**191**
Cx. (Mcx.) pleuristriatus/albipes							7																					**7**
Cx. (Mcx.) reducens								5																				**5**
Cx. (Mcx.) sp.	3	6	6	29	60						12																	**116**
Cx. (Mcx.) worontzowi					17	4	3	11		12	12	13	47	1	5	1		12	1					6		9	1	**155**
Cx. (Mel.) intrincatus														3														**3**
Cx. ocellatus		10		27	5		4			6	18	8	67	27	35	26	8	1	2	14	1	6	13	11		6	13	**308**
Hg. (Con.) leucocelaenus											6		29		1		8	2		3	7			4		9	2	**71**
Lu. (Lut.) bigoti																				1								**1**
Ru. (Run.) cerqueirai														7														**7**
Sa. (Sab.) purpureus																						3						**3**
Sh. fluviatile		4		32			4			7	15	8	45	28				2										**145**
Tr. pallidiventer			1					2		7	4	1	1		2									3	6			**27**
Tx. (Lyn.) theobaldi / moengoensis																								2				**2**
Tx. bambusicola aff						1																						**1**
Tx. guadeloupensis aff		2																										**2**
Tx. trichopygus aff					1																							**1**
Tx. purpureus/catharinensis				2	1																							**3**
Tx. sp.	3		2					1			3	1	1	2	1		1	1		1		2	2	2				**23**
Tx. trichopygus		1	2																									**3**
Wy. (Mim.) oblita															3	2												**5**
Wy. (Pho.) davisi			6	11				14		5	3	16	31	12	4	3	1	6				4	2			1	4	**123**
Wy. (Pho.) edwardsi			21					2			1																	**24**
Wy. (Pho.) pallidoventer		4	5	4	20		10			3		3	3		9													**61**
Wy. (Pho.) palmata													3															**3**
Wy. (Pho.) sp.		3		16							29																	**48**
Wy. (Pho.) theobaldi		38	2		26			40		26	8	3	9	17	3	8	2	5				10	1	3		2	1	**204**
Wy. (Spi.) airosai															1													**1**
Wy. (Wyo.) lutzi															9													**9**
Wy. personata															2									2				**4**
Wy. sp.															4													**4**
**Total**	**94**	**195**	**85**	**180**	**193**	**34**	**33**	**222**	**12**	**136**	**346**	**301**	**463**	**210**	**122**	**58**	**51**	**75**	**3**	**29**	**34**	**29**	**35**	**106**	**15**	**56**	**60**	**3124**
